# The Luck of the Draw: Wellcome's Institutional Fund for Research Culture

**DOI:** 10.12688/wellcomeopenres.20057.2

**Published:** 2023-11-20

**Authors:** Shomari Lewis-Wilson, Sonya Towers, Harriet Wykeham

**Affiliations:** 1 Wellcome Trust, London, England, UK

**Keywords:** Research Culture, Partial Randomisation, Research Funding, Grant-giving, Grant-making, Philanthropy

## Abstract

Wellcome's Institutional Fund for Research Culture (IFRC) closed call is an invite-only grant call in 2023. It is a departure from Wellcome's previous methods of institutional funding, providing institutions with up to £1m of grant funding to take on ambitious projects that advance research cultures and research environments that are equitable, diverse and supportive. Recognising the broad range of topics and ideas for advancing positive research cultures, IFRC is the first ever Wellcome-funding scheme to use partial randomisation to allocate funding. Applications were grouped by a funding committee into three categories (Gold, Silver and Bronze), with the applications selected for Gold being directly recommended for funding and all applications in the Silver group being set for funding by the randomiser. Applications grouped into Bronze were not funded. To ensure that this activity Wellcome's principles for openness and transparency, we have included the Python script for the call here. IFRC comes when efforts to fund positive and inclusive research cultures are mainstream. Similar efforts to support research culture activities at scale have come from the Research England Development (RED) Fund, and the next iteration of the Research Excellence Framework (REF 2028) will also mark 25% of the assessment criteria for people, culture and environment. IFRC was not designed with the national picture in mind but is a testament to Wellcome's values as an inclusive funder. The range of projects and geographies that IFRC will fund is exciting.Still, it also threw up several interesting social and philanthropic research questions we want to explore in future community-facing activities following the call. We hope that findings from IFRC projects become a valuable resource for institutions wishing to improve their research cultures and a catalyst for future change and discussion within the sector that makes academic careers more inclusive.

## Disclaimer

The views expressed in this article are those of the author(s). Publication in Wellcome Open Research does not imply endorsement by Wellcome.

## What is the Institutional Fund for Research Culture?

**Figure 1.**
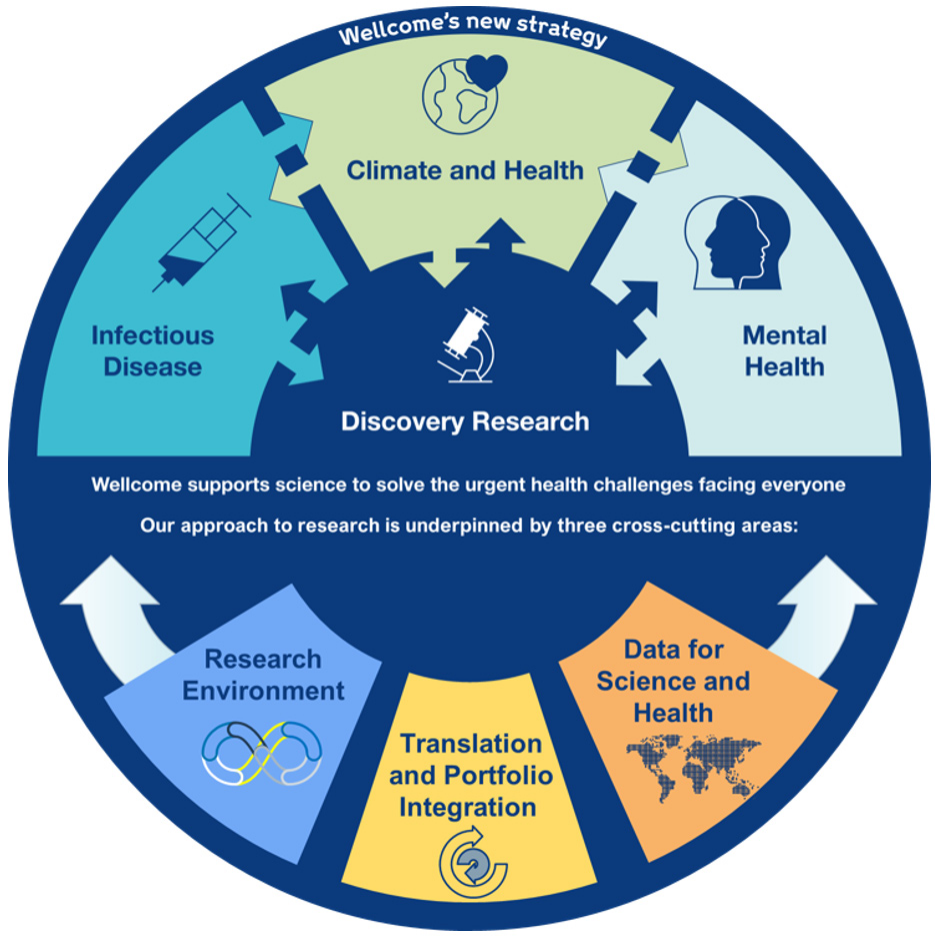
A schematic diagram presenting the Wellcome Trust Research Programme strategy that the Research Environment team cuts across. Adapted from Wellcome, Corporate Affairs by Carleigh Krubiner.

Wellcome’s Research Environment (RE) approach cuts across Wellcome’s strategy ^i^( Figure 1), considering how Wellcome funds research. It is grounded in three values:

- The research that Wellcome supports is strengthened by being ethical, open, and engaged.
- The people Wellcome funds thrive in equitable, diverse, and supportive cultures.
- Wellcome is an innovative, efficient, and inclusive partner and organisation.

Wellcome’s ongoing commitment to encouraging positive and inclusive research cultures ^ii^ and communities where everyone can thrive caters mainly to the second value. We utilise Wellcome’s **money, values, and influence** to change the academic research sector, enabling the researchers' Wellcome funds to thrive in positive and inclusive research environments.

Wellcome has a history of using “strategic” funding to support universities and other research institutions across the UK with activities such as supporting early career researchers, diversity and inclusion and public engagement ^iii^. However, our approach in the past to allocating the funding was not strategic, lacked specific outcome measures and favoured institutions with a strong track record of gaining Wellcome funding (you probably know who they are).

This got us thinking.

- Can we fund research culture activities at scale?
- How can we do this equitably, recognising that every research environment differs with various challenges, contexts, and available resources between institutions?
- How can we influence further positive developments in the research culture space, build trust and partner with the community to create solutions?

And so, the Institutional Fund for Research Culture (IFRC) was created.

We invited 43 institutions from the UK and the Republic of Ireland to apply for grants of up to £1m over two years. The invited organisations had to have held at least ten Wellcome grants in the last five years to be eligible. Funders, pharmaceutical companies, and other not-for-profit organisations were excluded. This shift from Wellcome’s previous approach meant we were targeting research institutions most likely to conduct research aligned with Wellcome’s strategic priorities ^iv^ and hosting researchers who could apply for Wellcome funding in the future. It also extended our reach of eligible institutions to all parts of the UK, which, viewed from an equity standpoint, broadened the opportunity for institutions with fewer resources to progress their research cultures as future centres of excellence. 

## How did we review the applications?

Applications were assessed according to the following criteria, guidance for which was shared online:

- **Identification of the barriers to a positive research culture and the potential to overcome them (25%)**
- **Breadth of impact (25%)**
- **Evaluation (20%)**
- **Institutional Commitment (15%)**
- **Team skills and experience (15%)**

**Figure 2.**
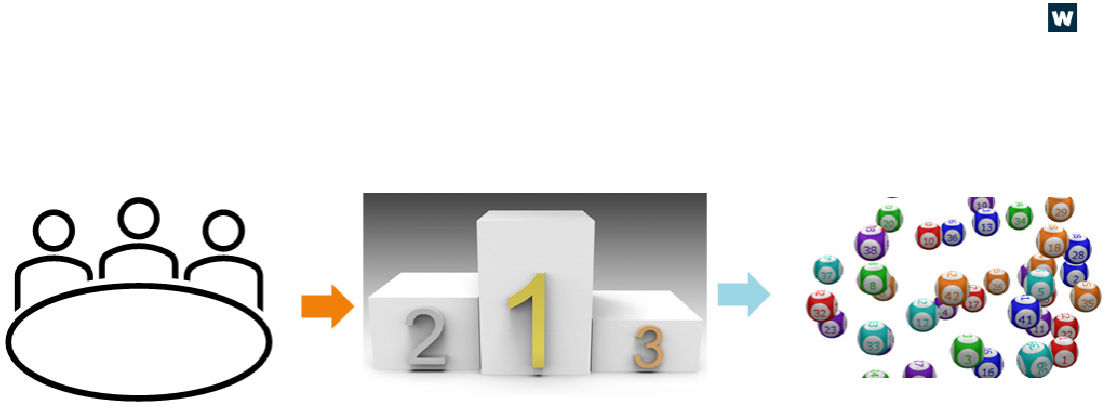
A simple schematic of the IFRC review process. Left: A committee assessed and scored the applications. Middle: Shows a podium representing how the committee grouped the applications into Gold, Silver, and Bronze. Right: Lottery balls representing the randomiser used for the silver applications.

The review process we used for this scheme is summarised in Figure 2. We recruited a funding committee of internal Wellcome colleagues and additional experts from other funders, research centres and higher education institutions in the UK, US, and mainland Europe. The committee scored applications as typical for a usual funding process, but rather than a ranked list, applications were sorted into groups of Gold, Silver, or Bronze, as agreed by the committee. Gold applications were exceptional and the top priority for funding. Applications in the Bronze group were not considered a priority for funding. Silver applications were fundable but with some risks or minor flaws. A **randomisation (lottery) process** using a Python script (a high-level programming language), the first of its kind in Wellcome history, decided which applications in this Silver group to fund. For transparency in this process, the code used for the randomisation can be accessed at the end of this article ^v^.

From the outset, we knew how difficult the challenge of assessing research culture would be. An institution that is typically well-funded could have more resources and tools to understand its research cultures than an institution with less funding. This could bias our process towards institutions with ‘bigger’ names or to projects claiming research culture change at “scale” but with not necessarily the most inclusive, brave, transformative, or thoughtful ideas. Now, suppose you have two institutions that got the same score, for example. How could we decide between Institution A’s project looking at research leadership capabilities and Institution B’s project looking at anti-ableism? Our intention for IFRC was not to tell the sector what “Wellcome” finds essential for positive research cultures. It was to help the sector find the answers for themselves, otherwise known as “equipping the problem solvers” ^vi^.

Within an application process, there are time constraints and limits to the information we could reasonably collect to base our assessment on. We also tried to be thoughtful and mindful of the questions that exist in the sector for how to devise suitable measures for research culture that consider context, the “distance travelled”, ^vii, viii^ and that this was a closed call to institutions with an already ‘warm’ relationship to Wellcome.

In this regard, we chose partial randomisation as the fairest funding mechanism, recognising that there is no “right” answer for research culture but plenty of solutions.

## Who did the funding go to?

The committee grouped 16% of applications into Gold, 56% Silver, and 28% Bronze. We funded 24 institutions in total. These are shown in Table 1 in no particular order. Though the collaborative grant between The University of Glasgow, the University of Edinburgh and the University of St Andrews was the only joined application of the call, some of the awardees will share their funding with institutions that were not invited to apply. These further beneficiaries are not named. 

**Table 1.** List of awarded institutions for Wellcome’s Institutional Fund for Research Culture 2023.

Organisation	Title
University of Strathclyde	Cultures of Collaborative Research in a Socially Progressive Technological University
King's College London	INKLUDE: Inclusion as a Norm at King's - Leadership, Understanding the issues, Developing individuals, Enacting change
University of Sheffield	WAARC: Wellcome Anti-Ableist Research Culture
Queen's University Belfast	NIRCI: Northern Ireland Research Culture Initiative (NIRCI)
University of Leicester	I-REACCH: Inclusive Research Environment Achieved through Culture Change
Newcastle University	Building Enhanced Leadership Capacity to Enable Psychologically Safe and Inclusive Research Environments
University of Durham	Reimagining Governance for a Flourishing Research Culture
Liverpool School of Tropical Medicine	LSTM – Promoting equitable partnerships
Institute of Cancer Research	Strengthening career pathways for technicians
University of Birmingham	ASPIRE: Access to Success Pathways: Inclusive Research Excellence (ASPIRE)
University College London	Enabling collaboration and team science
University of Oxford	Leading Across Boundaries: Researcher-Driven Leadership Development
University of Nottingham	REC-HURDLEs: Revisiting Employment Contracts and Helping Under-represented Researcher groups to Develop, Lead and Excel
University of East Anglia	Creating a Culture of Inclusion: Increasing Diversity and Equity of Access - IDEA
University of Glasgow, University of Edinburgh & University of St Andrews	One Collegial Framework for many contexts: Testing a new model for Creating culture change
University College Cork	The External Voice in Defining an Engaged and Inclusive Institutional Research Culture: Engaged & Inclusive Research Culture Alliance (ERICA)
Birkbeck University of London	Research Culture at Birkbeck
Lancaster University	Reimagining Research Practices: Towards a sustainable, ethical and Inclusive Future
University College Dublin	RENEEW - Research Culture Networking, Empowerment, Engagement, and Wellbeing
University of Dundee	Progressing positive and inclusive research culture at the University of Dundee
University of York	Valuing Voices for Equitable and Responsible Research
Cardiff University	Ignite: Research Culture at Cardiff University

## What we hope to achieve with IFRC

We are excited about the range of projects and geographies that IFRC will fund. We hope the funding is viewed as another strong example of Wellcome using its **money, values, and influence** to affect positive change in the external research sector. For example:

- **Money:** Investment in diverse research culture ideas and institutions helps Wellcome and the research sector develop and share evidence about approaches that work or don’t. The projects benefit researchers who may apply to Wellcome for funding of their own in future.
- **Values:** Wellcome’s **brave, transformative, inclusive, and thoughtful** use of partial randomisation adds to the evidence base for where it makes sense to use this funding mechanism strategically. In this case, using a randomiser helped remove bias and the need for consensus in the final recommendations. We would probably *not* find the use of a randomiser appropriate for an open-mode grant going to an individual where Wellcome wants to have more of a say on what it considers the most ‘bold creative and high-quality ^ix^’ applications to be. Nonetheless, a trial comparing the success of applicants selected by a standard process versus a randomiser would be fascinating, as discussed in this article ^x^ and this blog ^xi^.
- **Influence:** The projects succeed and influence more positive research culture and practice change in the UK and beyond. Wellcome enables idea and knowledge sharing as a partner through research culture community events, report-writing, and tool development, which transmits good practice beyond the lifetime of the grants.

The call has also raised several exciting social and philanthropic research questions we want to explore, evaluate, and engage the sector with. We will invite everyone who applied to join a “community of practice” (CoP) over the next two years. Within this community, we want to examine several themes and questions, such as:

- How do we promote collaboration, sharing and transparency of different ideas and approaches within the research culture space?
- What does success look like for the call, and how should we evaluate this?
- What commonalities and cross-cutting themes exist between the awarded applications?
- What can we learn from institutions about the existential challenges of implementing culture change at scale?
- When the funding finishes, what has worked and what hasn’t, and what, if anything, does this say about the decision-making we did and didn’t do for this scheme?
- How can we utilise the outputs of this call to create a permanent shift in institutional research cultures for the future?

We look forward to sharing more soon as we build this exciting programme. If you have any questions, please reach out to researchenvironment@wellcome.org

## Data Availability

No data are associated with this article. Zenodo: IFRC Award Allocator, https://doi.org/10.5281/zenodo.10043374 ^xii^. This project contains the following extended data: Award_allocator.py (This code simulates allocating funds to grant applications in a random order, as Wellcome used for its IFRC scheme) Data are available under the terms of the Creative Commons Attribution 4.0 International license (CC-BY 4.0).
