# Supplementary material for: The Luck of the Draw: Wellcome's Institutional Fund for Research Culture
Source: Wellcome Open Res. 2023 Nov 20;8:525. Originally published 2023 Nov 13. [Version 2] doi: 10.12688/wellcomeopenres.20057.2 (PMC10869953; doi:10.12688/wellcomeopenres.20057.2)
Supplement: Peer review history

# Reviewer report: Reviewer response for version 2

**DOI:** 10.21956/wellcomeopenres.22730.r72481

**Published:** 2024-02-15

**Authors:** Alejandra Recio Saucedo

**Affiliations:** 1 University of Southampton, Southampton, England, UK

In this open letter the authors report the use of partial randomisation to allocate funds for the Wellcome's Institutional Fund for Research Culture (IFRC) closed call. The report is written in an engaging way and provides details for the reasons to use this method to make funding decisions. The potential for the use of randomness in funding allocation decisions has been recognised (e.g., Avin, 2018) ^1^ and it is quite positive to see an organisation identifying a call for which the combination of peer review and technology in making funding decisions was found to be appropriate and implemented.

 Overall the information in the letter provides detail about the process that was followed and the steps that will be taken to assess the impact of grants that received funds, which may be used to build a case around a more widespread use of the method. It is also commendable and a great contribution to transparency that the authors have published the python code used in the randomisation stage.

 There are four areas where additional information would be of great value:

**1. **The authors wrote: “our approach in the past to allocating the funding was not strategic, lacked specific outcome measures and favoured institutions with a strong track record of gaining Wellcome funding”Is this the view of Wellcome or the authors’ assessment of some areas of opportunity for the funding organisation? If the former, a reference would be helpful to add. If the latter, it may be important to add detail of the evidence that supports  the statement. This is because lacking specific outcome measures in funding allocation would be a vital area of development for an organisation, and I find it difficult to understand how these were not in place. In addition, one could argue that publishing figures of how funds were distributed in terms of number of awards/grants, number of researchers, etc. are a form of outcome measure, which are available for most funding organisations.

**2. **On the eligibility criteria for the IFRC and fairness.What is the likelihood that institutions with fewer resources have had at least ten Wellcome grants in the last five years? Might this criterion have inadvertently excluded some institutions from being eligibility for the call? The use of partial randomisation was geared towards increasing fairness. Was this a consideration throughout the decision process? Were all institutions that met the criteria of at least 10 Wellcome grants in the last five years invited? Or was there a selection process? Providing information about the invitation process would answer some of the questions.

**3. **A number of factors supporting the adoption of partial randomisation to allocate research funds have been discussed, some of them being about removing potential bias that may affect decisions and others being about increasing efficiencies. The reasons to use partial randomisation provided in the text (“In this regard, we chose partial randomisation as the fairest funding mechanism, recognising that there is no “right” answer for research culture but plenty of solutions”) cover the aspect of increasing fairness, which can be argued addresses bias. For the second factor, was there any insight on the use of the IFRC Award Allocator addressing time constraints and increased efficiencies? This would be of great interest considering that the process reported a more traditional review of applications that required a committee scoring and sorting proposals. Furthermore, some additional information on the criteria for scoring and sorting would be valuable as it is part of the partial randomisation process, and would need to be reported as was the python code.

**4. **Referencing differing views and opinions. Random allocation in funding has been implemented by various organisations (e.g., the Health Research Council of New Zealand (HRC), the Volkswagen Foundation in Germany and the Swiss National Science Foundation). The article by Liu et al (2020) ^2^ report the views of researchers on the use of this method at (HRC), and as we could anticipate, not everybody agrees with its use. As such, it would be good to see the way that the IFRC addressed the challenges faced when implementing this method, as I believe there must have been diverse opinions in the use of partial randomisation. I personally would find it highly valuable to learn the opinion of researchers, funding committees and the general public on the use of this method and how it is appropriate in some funding calls for reasons of fairness and efficiency. Furthermore, it would be of interest to learn how the method ensures that the best research (from a scientific, relevance, innovation, and potential for delivering benefit points of view), is still supported when using partial randomisation. I recognise that the first stage in selecting awards addresses these areas, but it would be helpful to have more details on this reported in the letter.

 I look forward to reading further reports, letters and articles on this topic.

Does the article adequately reference differing views and opinions?

No

Are all factual statements correct, and are statements and arguments made adequately supported by citations?

Yes

Is the Open Letter written in accessible language?

Yes

Where applicable, are recommendations and next steps explained clearly for others to follow?

Yes

Is the rationale for the Open Letter provided in sufficient detail?

Yes

Reviewer Expertise:

Research on research; EDI and academic policy engagement; health services research

I confirm that I have read this submission and believe that I have an appropriate level of expertise to confirm that it is of an acceptable scientific standard, however I have significant reservations, as outlined above.

## References

[1] : Mavericks and lotteries. *Stud Hist Philos Sci* .2019;76: 10.1016/j.shpsa.2018.11.006 13-23 10.1016/j.shpsa.2018.11.006 31558205

[2] : The acceptability of using a lottery to allocate research funding: a survey of applicants. *Res Integr Peer Rev* .2020;5: 10.1186/s41073-019-0089-z 3 10.1186/s41073-019-0089-z 32025338 PMC6996170

# Reviewer report: Reviewer response for version 2

**DOI:** 10.21956/wellcomeopenres.22730.r69985

**Published:** 2024-02-09

**Authors:** Anne-Marie Coriat, Jennifer Cusiter

**Affiliations:** 1 Registrar, College of Medicine and Veterinary Medicine, The University of Edinburgh (Ringgold ID: 3124), Edinburgh, Scotland, UK; 2 Head of Research Office, College of Medicine and Veterinary Medicine, The University of Edinburgh (Ringgold ID: 3124), Edinburgh, Scotland, UK

This is an exciting initiative and the authors are to be commended both on launching the initiative and in particular for publishing the methodology and sharing their experience. This is excellent to see.

1. **The rationale for the Open Letter** is clear and in sufficient detail
2. **Does the article adequately r** **eference differing views and opinions**.

The articles cited by Gadd et al (vi) and Curry et al (viii) provide useful background and the opening paragraphs of the letter set the scene for the principles and rational for the call. These are clear. However, this is not as wide a contextual setting as might be helpful. In setting out how they might draw conclusions from this very welcome experimental approach some additional context would be helpful to:

  

- illustrate both the nature of culture as a concept and differences in how this is experienced. It is important to set out the logic for the role that policy and funding may play in change (both positive and negative) and to understand how reviewers were briefed, trained and supported to deliver this vision; there is no justification as to why the call was invite only.
- frame how learnings from related Wellcome Trust investments e.g. institutional translational partnerships, PhD programmes and by those of other funders might have informed the design of the call and the overall approach including for example work included in links 1, 2, 3 and 4 at the end of this report.
- acknowledge that the system in which this call is operating is inherently dynamic and complex, assumptions are hard to generalise. The operating context across the UK is not equal, nor as the authors highlight is it equal at an institutional level. For example:
  - Whilst the authors are correct that ‘typically well-funded (institutions) could have more resources to understand their research cultures the assertion that this means change it is easier to affect may not be correct, with more traditional operating models in place.
  - Whilst it is absolutely true that discussion of culture and the importance of a thriving research ecosystem is more mainstream than it was across the UK 5 years ago, baseline operating contexts are very different across the devolved nations. It is important to acknowledge this in particular in relation as to how applications are reviewed against different baselines, an extremely difficult task, hence the importance of experiments like this! For example, whilst its absolutely true that RED(F) funding has delivered significant opportunities this is England only and that whilst REF has a UK wide 4 nation approach at a process level, funding allocations are allocated/calculated differently by the various devolved governments at a National level.

 

- The authors state that they hope that findings from IFRC projects become a valuable resource for institutions wishing to improve their research cultures and a catalyst for future change and discussion within the sector that makes academic careers more inclusive. We hope also that this will be the case. We would suggest also that experiments like this are crucially important for Funders to reflect on their own ways of working, to understand how the way they work drive behaviours and cultures. Experiments such as this are, we would argue, a crucially important opportunity for co-creation of future research and policy at a Funder, national and institutional level

**3. Is the article written in open and accessible language** **? ** Yes

**4. Next steps.**  This is an exciting experiment, we are delighted to see it happen, and for the letter to be shared so openly. In terms of the questions the authors posed before creating the IFC

- Can they fund at scale?
- Can they fund equitably?
- Can they influence further positive developments?

The commitment to building a community of practice to explore the next steps is incredibly valuable and we fully support this, a few points of clarification would be helpful in terms of:

- Whether by scale the authors mean breadth of impact rather than / or in addition to scale of funding?
- In terms of equitable ways of working it would be helpful to acknowledge that it would be almost impossible to understand all nuances, or to remove all bias – but stress the importance of understanding and acknowledging baseline positions. Institutional ways of working and operational factors play a significant role in equity. Work by the University of Oxford from 2022 on equity in research funding is a valuable and useful read and sets out the issues well.
- Influencing further positive developments.  The applicants state that from the outset, they knew how difficult the challenge of assessing research culture would be and that they want to understand what has worked and what hasn’t. To an extent this will always be context dependent and always messy. Setting the right conditions and behaviours that encourage learning and continual improvement across all parts of the system are likely to be as (if not more) important than policy. How will Wellcome itself build a learning approach into their own ways of working and make the best use of this and other experimental approaches?
- In terms of influence, are Wellcome collaborating with other funders UK or globally to join up across the funder community of practice.

In conclusion we are delighted to see this experimental approach and commend Shomari Lewis-Wilson, Sonya Towers and Harriet Wykeham for submitting this open letter.

 A few references that may be helpful:

1. ref [1]
2. The experimental research funder’s handbook (2nd edition) (figshare.com)
3. Emerging Research Cultures – Resources (wordpress.com)
4. Equity and Inclusivity in Research Funding (ox.ac.uk)

Does the article adequately reference differing views and opinions?

Partly

Are all factual statements correct, and are statements and arguments made adequately supported by citations?

Yes

Is the Open Letter written in accessible language?

Yes

Where applicable, are recommendations and next steps explained clearly for others to follow?

Yes

Is the rationale for the Open Letter provided in sufficient detail?

Yes

Reviewer Expertise:

Areas of expertise include - Research Policy, Research Funding, Research Careers , Research Culture, HE policy, University Operations and Strategy

We confirm that we have read this submission and believe that we have an appropriate level of expertise to confirm that it is of an acceptable scientific standard.

## References

[1] : Setting the right tone. *Elife* .2020;9: 10.7554/eLife.55543 10.7554/eLife.55543 32036857 PMC7010405

# Reviewer report: Reviewer response for version 2

**DOI:** 10.21956/wellcomeopenres.22730.r72483

**Published:** 2024-01-27

**Authors:** Krist Vaesen

**Affiliations:** 1 Eindhoven University of Technology, Eindhoven, Netherlands Antilles

The authors report on a new funding initiative of Wellcome, namely Wellcome’s Institutional Fund for Research Culture (IFRC). I would like to approve the paper, but recommend the authors to clarify a couple of things.

 Although the paper is written in accessible language, it is sometimes hard to follow. The article assumes more background knowledge than one can expect the general reader to have. For instance, the first sentences in the main text directly dive into Wellcome’s Research Environment, without explaining what this environment exactly is (some readers might not even know what Wellcome is). Figure 1 is meant to illustrate something, but it is not clear exactly what. Without further explanation (for instance in the Figure’s caption), the reader, I fear, will find the figure more confusing than illuminating.

 Other points in need of clarification include:

-  it is suggested that Wellcome has a history of strategic funding (p. 3), but also that it doesn’t (p. 4)
-  the reasons for working on-invitation only (doesn’t this conflict with inclusivity?)
-  the types of projects (which topics?) that IFRC has funded.
-  the evaluation criteria used to assess applications (for instance: one criterion is called “Evaluation”; evaluation of what?)

Given these clarifications, the paper will be a valuable addition to the literature on research funding. I, at least, am curious about the further development of the IFRC, and about the outcomes of the projects that it has funded.

Does the article adequately reference differing views and opinions?

Yes

Are all factual statements correct, and are statements and arguments made adequately supported by citations?

Partly

Is the Open Letter written in accessible language?

Partly

Where applicable, are recommendations and next steps explained clearly for others to follow?

Yes

Is the rationale for the Open Letter provided in sufficient detail?

Yes

Reviewer Expertise:

Philosophy of science, meta-science, research policy

I confirm that I have read this submission and believe that I have an appropriate level of expertise to confirm that it is of an acceptable scientific standard, however I have significant reservations, as outlined above.
